# Case Report: CLAPO syndrome: a case management and literature review

**DOI:** 10.3389/fped.2026.1867411

**Published:** 2026-07-08

**Authors:** Liang Wang, Ming Wang, Xiaojuan Li, Bingxuan Jiao, Dan Song

**Affiliations:** 1 Department of Vascular Anomalies and Interventional Radiology, Children’s Hospital Affiliated to Shandong University, Jinan, China; 2 Department of Vascular Anomalies and Interventional Radiology, Ji’nan Children’s Hospital, Jinan, China; 3 Department of Hospital Office, Qilu Hospital of Shandong University Dezhou Hospital, Dezhou, China

**Keywords:** capillary malformation, CLAPO syndrome, lower lip, management, review

## Abstract

**Background:**

CLAPO syndrome, a condition potentially associated with the PIK3CA-related overgrowth spectrum, is a rare vascular malformation that was first reported in 2008 and formally incorporated into the ISSVA classification in 2018.

**Objective:**

This study intends to investigate the clinical features of CLAPO syndrome.

**Methods:**

We report one clinical case of CLAPO syndrome admitted to our hospital and retrospectively summarize findings of all previously published relevant cases.

**Results:**

Together with the current case in this study, a total of 31 patients across 11 publications were enrolled for analysis. All 31 patients exhibited lower lip capillary malformation (CM). Additionally, lymphatic malformations were observed in 25 cases, venous malformations in 17 cases, and varying degrees of tissue overgrowth in 10 patients.

**Limitations:**

Due to the extremely low incidence of this disease, the sample size is relatively small. Further studies are required to clarify its pathogenesis, therapeutic efficacy and genetic characteristics.

**Conclusion:**

Lower lip CM serves as a predominant manifestation of CLAPO syndrome. Clinicians should raise the suspicion of CLAPO syndrome in patients presenting with lower lip CM. Notably, enlarged venous vessels visible on the median raphe of the tongue may also be regarded as a clinical feature of CLAPO syndrome.

## Introduction

CLAPO syndrome is a rare overgrowth disorder characterized by capillary malformation of the lower lip (lower lip CM), predominant lymphatic malformation in the cervicofacial region, asymmetric facial features, and partial or generalized overgrowth. First observed in 2008 (1) and officially included in the ISSVA classification in 2018, CLAPO syndrome has been associated with somatic activating mutations in the PIK3CA gene (2). In this report, we present a case of CLAPO syndrome from our institution and summarize published cases to explore the clinical characteristics of the syndrome and enhance our understanding of it. This study was approved by the ethics committee of Children's Hospital Affiliated to Shandong University.

## Case report

**Figure 1:**
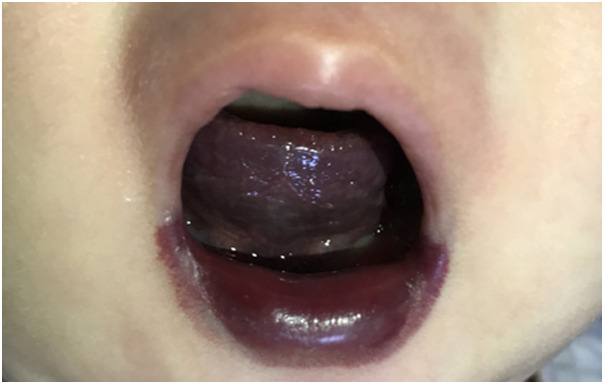
An erythematous-violaceous macule of the lower lip and tongue.

A 46-day-old full-term female infant delivered by cesarean section with a birth weight of 3.6 kg was admitted to our hospital due to a capillary malformation of the lower lip. The lesion extended across the vermilion border and presented symmetrical distribution. No similar lesions were observed in her parents or siblings (a 16-year-old brother and a 4-year-old sister). Physical examination showed an erythematous-violaceous macule involving the lower lip and tongue, with no soft tissue overgrowth (Figure 1). Magnetic resonance imaging (MRI) showed scattered vascular malformations involving the lower lip, tongue, mandibular region, and anterior cervical region. The child received sclerotherapy with pingyangmycin and polidocanol, resulting in a noticeable fading of the lesion's surface color upon follow-up.

**Figure 2:**
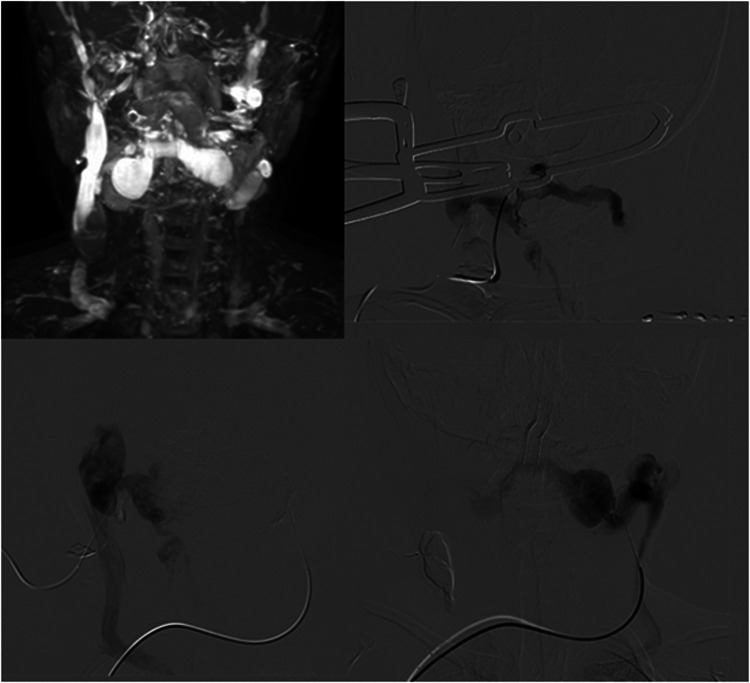
A gradually enlarged venous vessel turned visible on the median raphe of the tongue and drains bilaterally into the large vessels of the cervical region.

**Figure 3:**
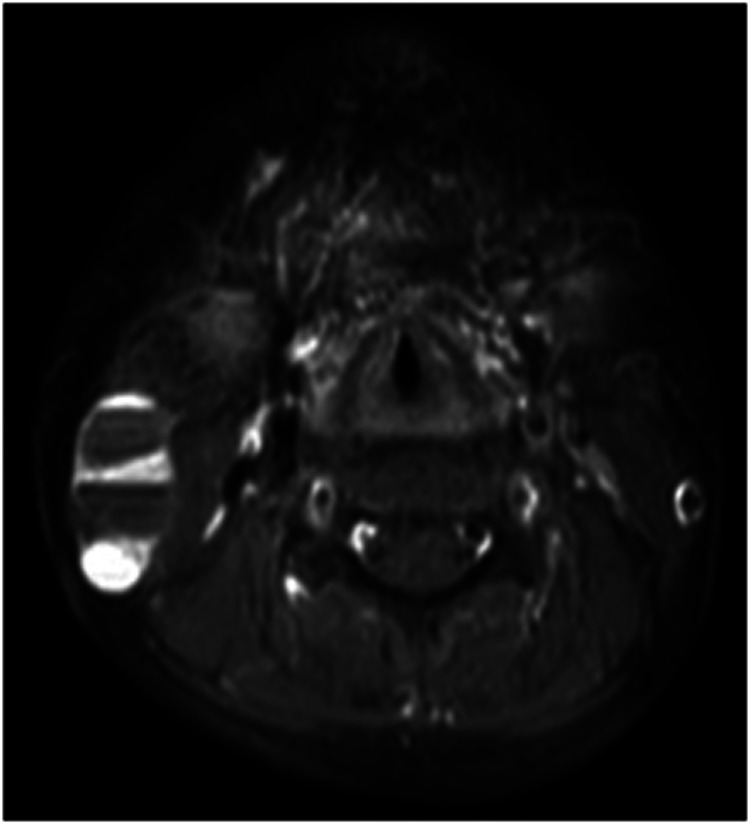
Lymphatic malformation with intracapsular hemorrhage.

At 1 year old, she developed overgrowth in the mandibular region on the right side of her face. She underwent four additional sclerotherapy procedures over the next 2 years without complications. During sclerotherapy, a gradually enlarging venous vessel was observed on the median raphe of the tongue, draining bilaterally into the large vessels of the cervical region, as seen on DSA angiography and MRI (Figure 2). At 3 years and 6 months, a 2 cm diameter mass suddenly appeared in her right neck, which ultrasound and MRI indicated as a lymphatic malformation with intracapsular hemorrhage (Figure 3). Ultrasound-guided extraction of the lymphatic sac effusion (6 mL) was followed by injection of 1 mg of pingyangmycin for sclerotherapy. She has not presented any neurological symptoms or intellectual disability up to age 4. Unfortunately, genetic testing was recommended for the child, but her parents did not agree to the test due to the cost.

## Discussion

**Figure 4:**
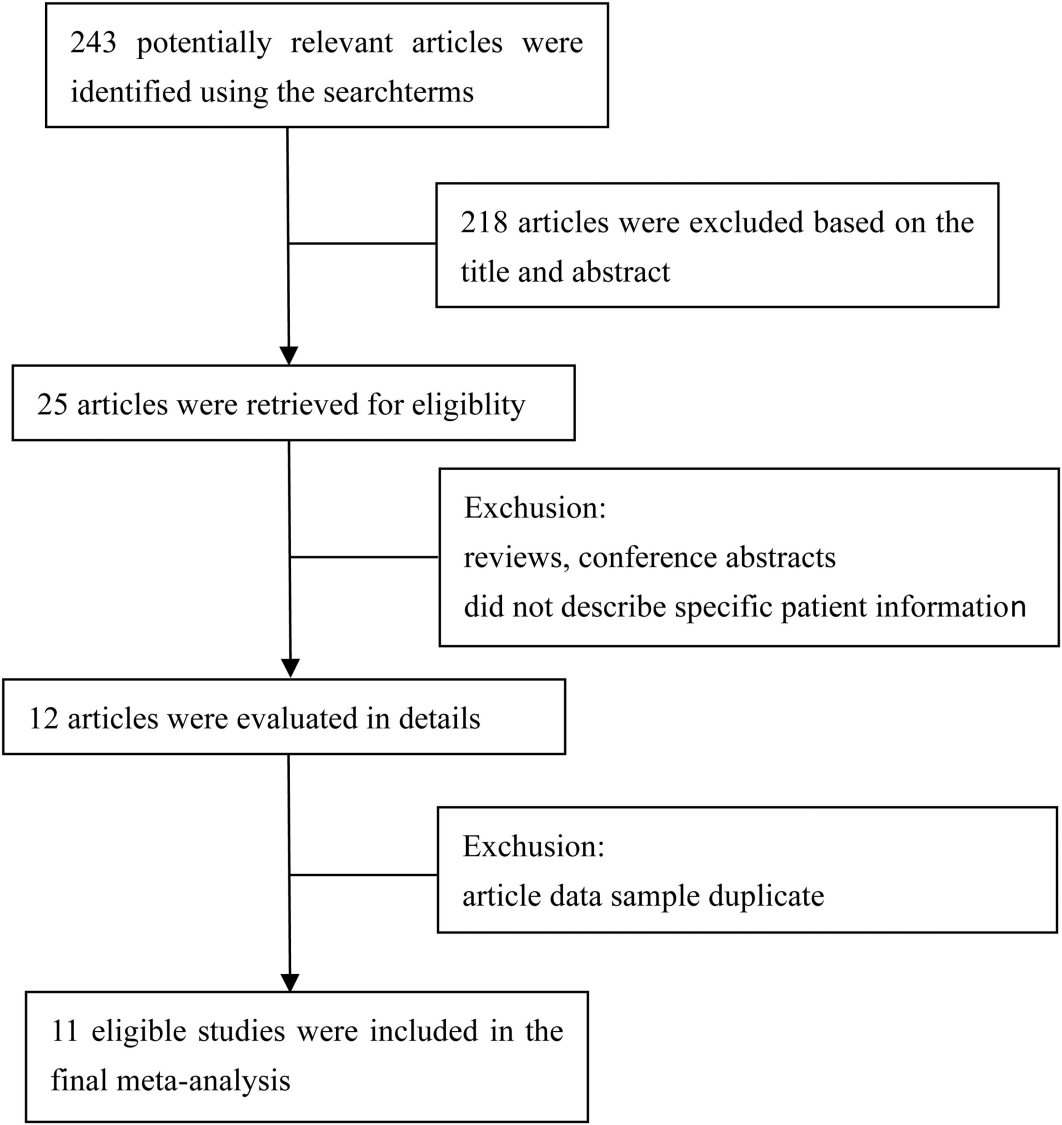
Flowchart of the study selection process.

CLAPO syndrome is a rare vascular malformation with few reported cases in the literature. A PubMed search was conducted using the terms “lower lip, capillary malformation” and “CLAPO”, and only English-language articles with full-text availability were included (Figure 4). The search covered all entries published from 2008 to the present. The results were verified by two independent authors to exclude duplicate papers. Overall, 13 publications were excluded, including 4 reviews, conference abstracts, and other articles that did not describe specific patient information. Additionally, the cohort of 13 patients reported by Rodriguez-Laguna L (2) included the six initial samples reported by López-Gutiérrez JC (1). Together with the case in this study, a total of 11 publications (2–12) involving 31 patients were included. Clinical findings are summarized in Table 1. The age of the included subjects ranged from 1 day to 58 years at the time of reporting, with 20 females and 11 males. There was no family history of similar lesions (3, 4, 7), and no psychomotor delay or intellectual deficits (2–5) were evident in the CLAPO patients.

**Table 1:** Reported cases of CLAPO syndrome.

Literature	Age	Gender	CM	LM	VM	overgrowth
Krämer D 2016 (3)	12y	F	lower lip	tongue and floor of the mouth	lower lip, chin, and anterior cervical region	mandibular region of the face, neck, and left side of the thorax
Flores-Terry MÁ 2018 (4)	2y	M	lower lip	none	none	right hemihypertophy of leg
Rodriguez-Laguna L 2018 (2)	40y	M	lower lip, right area of the body, reddish color	bilateral lymphedema with predominance of foot-knee	multiple varicose veins in the legs	partial overgrowth of the right side of the body
	17y	F	lower lip, left inguinal region	lower limbs	none	left leg and right arm hypertrophy
	20y	M	lower lip	tongue and mandibular region	neck	generalized
	20y	F	lower lip	tongue and mandibular region	neck	none
	16y	F	lower lip	tongue and mandibular region	neck	none
	11y	F	lower lip, neck	tongue and mandibular region	neck	none
	18y	F	lower lip	intraoral part of the lip	none	none
	12y	M	lower lip, right glute	cervicofacial region, tongue, gluteus	neck	none
	4y	F	lower lip, multiple, extensive: right lower limb, face, neck	none	neck	none
	2y	F	lower lip, left hand, between the 4th and 5th fingers	cervicofacial region, tongue	neck	segmental hypoplasia
	5y	M	lower lip, glabella and neck	cervicofacial region	neck	none
	4y	M	lower lip, tongue and right ankle	cervicofacial region, tongue	neck	none
	7y	F	lower lip, hip	tongue and mandibular region	tongue, gingiva, left hip	none
De M H 2018 (5)	18m	M	lower lip, neck, jaw, and scalp	none	none	none
Downey C 2018 (6)	13y	F	lower lip	tongue	NR	none
González-Hermosa MR 2019 (7)	16m	M	lower lip	head and neck	the median raphe of tongue	facial asymmetry
Jimenez-Cauhe J 2021 (8)	18y	F	lower lip	NR	NR	none
	21y	F	lower lip	tongue	NR	none
Cerejeira D 2021 (9)	18y	F	lower lip	lower lip, tongue	none	none
	20y	F	lower lip	oral lip	none	none
	58y	F	lower lip	none	neck	none
	22y	F	lower lip	oral lip, tongue, chin	none	face, left neck
	51y	M	lower lip, forehead, dorsum nasi, philtrum	tongue	tongue	none
	37y	F	lower lip	oral lip	none	none
	32y	F	lower lip	none	none	none
Moreno Alfonso JC (10)	8m	M	lower lip	present, but location was NR been reported	none	face, toe
Tran AX (11)	1d	M	lower lip	neck	none	none
Hewitt N (12)	1m	F	lower lip	neck	neck	NR
Current case	4y	F	lower lip	neck	tongue, mandibular, neck	facial asymmetry

y, years; m, months; d, days; M, male; F, female; CM, capillary malformation; LM, lymphatic malformation; VM, venous malformation; NR, not report.

One of the defining characteristics of CLAPO syndrome is the presence of lower lip capillary malformation (CM) in all 31 patients. Rodriguez-Laguna L described three distinct patterns of midline lower lip CM (2): a narrow midline CM with a reddish color, a CM involving the entire lower lip with a predominant brown/purple color, and a CM including the central inner area of the lower lip without external skin involvement beneath the vermilion. In addition to the lower lip CM, capillary malformations can also appear in areas such as the tongue, jaw, face, and neck.

Lymphatic malformation is observed in 25 of the 31 patients, commonly involving the lip, oral mucosa, neck, and tongue. Unlike lower lip CM, which is present at birth, lymphatic malformations may remain undetected until symptomatic onset, as demonstrated in cases #4, #17, and #28. Additionally, 17 cases with varying degrees of venous malformation were reported. Notably, progressively enlarged venous vessels located on the median raphe of the tongue and draining bilaterally into large cervical vessels, may be a clinical feature of CLAPO syndrome, similar to case #18 (7). Overgrowth, which tends to be segmental and rarely generalized in CLAPO syndrome (2), was present in 10 of the 31 reviewed cases.

Due to the rarity of CLAPO syndrome, it is often underdiagnosed, and there is no standard treatment. Patients treated with pulsed dye laser for capillary malformation experienced significant reduction in erythema and fewer side effects (9). Sclerotherapy has demonstrated good clinical efficacy in the treatment of vascular malformations, including lymphatic and venous malformations, and is gradually becoming the first-line minimally invasive treatment (13, 14). In our case, sclerotherapy achieved satisfactory results in both malformed vascular lesions and lymphatic malformation. Additionally, sirolimus, which targets the mTOR pathway, was first used to treat Kaposiform Hemangioendothelioma in 2010 (15); subsequent reports have demonstrated its promising therapeutic potential in vascular abnormalities (16–19), showing that it can reduce lesion volume and improve clinical symptoms and quality of life (17, 20), and it has gradually become one of the treatment options for vascular abnormalities. In González-Hermosa MR's report, a neonate (born at 34 weeks) who was treated with sirolimus for 13 months showed a notable decrease in cervicofacial lymphatic malformations (7).

Given that CLAPO syndrome may be associated with the PIK3CA-related overgrowth spectrum, future experiences with sirolimus treatment may provide further insights.

Despite summarizing all published cases in this paper, the number of patients is limited due to the rarity of the disease and our incomplete understanding of CLAPO syndrome. Further research is needed to explore treatment experiences and genetic information.

## Conclusion

Patients with CLAPO syndrome almost always present with lower lip CM, and lymphatic malformations, with or without concomitant venous malformations, may become evident after the neonatal period or be present at birth, while overgrowth may not be particularly obvious. Clinicians should heighten awareness of the possibility of CLAPO syndrome in patients with lower lip CM. Additionally, the presence of an enlarged venous vessel on the median raphe of the tongue may also be a clinical feature of CLAPO syndrome.

## Data Availability

The original contributions presented in the study are included in the article/supplementary material, further inquiries can be directed to the corresponding author.
